# Exploring the relationships among teacher–student dynamics, learning enjoyment, and burnout in EFL students: the role of emotional intelligence

**DOI:** 10.3389/fpsyg.2023.1329400

**Published:** 2024-01-08

**Authors:** Ying Li, Li Zhang

**Affiliations:** School of Foreign Languages, Xinjiang Normal University, Ürümqi, China

**Keywords:** teacher–student relationship, learning enjoyment, emotional intelligence, burnout, EFL students, structural equation modeling (SEM)

## Abstract

**Introduction:**

Within the realm of Chinese English as a Foreign Language (EFL) education, this research endeavors to explore the intricate interplay among teacher-student relationships, learning enjoyment, and burnout. It specifically aims to investigate the potential mediation role of emotional intelligence. The study delves into the experiences of 806 EFL students to comprehensively scrutinize these dynamics.

**Methods:**

Employing Structural Equation Modeling (SEM), this study conducts a thorough analysis of the relationships between teacher-student dynamics, learning enjoyment, and burnout among EFL students. The primary objectives involve uncovering correlations among these factors and elucidating the potential mediating impact of emotional intelligence.

**Results:**

The findings underscore robust associations between positive teacher-student relationships, heightened learning enjoyment, and reduced levels of burnout among EFL students. Noteworthy is the pivotal role of emotional intelligence, acting as a mediator, offering insights into the intricate ways in which teacher-student relationships and learning enjoyment influence burnout levels.

**Discussion:**

These outcomes highlight the significance of nurturing students’ emotional intelligence as a protective factor against burnout, advocating for tailored educational interventions. The research advocates for proactive measures to enhance emotional intelligence among EFL students, emphasizing its potential to mitigate burnout. Moreover, it suggests pedagogical strategies and institutional support prioritizing emotional intelligence to foster the well-being and academic success of Chinese EFL students.

## Introduction

In the current fast-paced educational domain, the well-being of students stands as a paramount concern. The escalating pressures of academia, coupled with personal challenges, have propelled student burnout into the spotlight (Teuber et al., 2021; Salmela-Aro et al., 2022). This state of physical and emotional exhaustion, often accompanied by detachment and diminished personal accomplishment, wields profound influence over students’ academic performance, mental health, and overall quality of life (Maslach et al., 2001; Kim et al., 2018).

Student burnout, a complex interplay of various factors, is shaped by academic demands, social pressures, and personal hurdles (Kim et al., 2018; Loi and Pryce, 2022). It manifests in deep-seated exhaustion, cynicism, and reduced sense of achievement (Maslach et al., 2001; Madigan and Curran, 2021), presenting in forms ranging from disengagement with coursework to declining academic prowess and even mental health concerns (Tang et al., 2021; Liu, 2023). The ramifications of student burnout ripple far beyond the classroom. Academically, it leads to plummeting grades, heightened dropout rates, and stalled career trajectories (Salmela-Aro and Read, 2017). Beyond academia’s confines, it fuels emotional distress, depression, and anxiety (Madigan and Curran, 2021). Given these profound implications, delving into the antecedents and mediators of student burnout becomes ever more imperative.

One potent variable in this equation is the quality of teacher–student relationships (Roorda et al., 2017; Taxer et al., 2019). These relationships stand as a linchpin of the educational experience, offering students a haven of engagement, security, and emotional sustenance (Pianta, 1999; Gastaldi et al., 2014; Longobardi et al., 2018). Conversely, strained or negative relationships breed stress, detachment, and emotional depletion – all fuel for burnout (Hakanen et al., 2006). While empirical evidence underscores the impact of teacher–student relationships, the intricate mechanisms underlying this connection invite further exploration.

Emotional intelligence, as envisioned by Salovey and Mayer (1990), encompasses the ability to recognize, comprehend, and manage emotions in oneself and others, steering how individuals navigate intricate webs of interpersonal relationships and emotional landscapes (Salovey and Mayer, 1990; Abe, 2011; Petrides and Mavroveli, 2018). As students progress through their educational odyssey, their emotional intelligence may wield influence over their capacity to cope with stressors, regulate emotions, and maintain a positive perspective in the face of challenges (Brackett et al., 2010; Molero Jurado et al., 2021). Emotional intelligence is posited as a pivotal mediator in the interplay between teacher–student interactions and student burnout (Chamizo-Nieto et al., 2021; Shariatpanahi et al., 2022). It is hypothesized that positive teacher–student relationships may nurture the growth of emotional intelligence, equipping students with the tools to adeptly navigate stressors and challenges (Saklofske et al., 2012; Chamizo-Nieto et al., 2021), ultimately mitigating the risk of burnout.

Another pivotal facet of interest in this study is learning enjoyment (Frenzel et al., 2009; Dewaele and Dewaele, 2020). When students derive pleasure from their learning experiences, they are more likely to be intrinsically motivated, engaged, and emotionally invested in their studies (Dewaele et al., 2023). Learning enjoyment closely aligns with the Self-Determination Theory (SDT; Ryan and Deci, 2000), asserting that individuals are propelled when activities align with their values and are perceived as enjoyable and engaging. This intrinsic motivation and enjoyment stand as bulwarks against burnout, a state characterized by disengagement and emotional depletion (Wu et al., 2023).

Moreover, the design of the study serves to contribute meaningfully to the existing educational literature by adopting a comprehensive approach to untangle the intricate web of variables influencing student burnout. The utilization of SEM as the primary data analysis method allows for a nuanced understanding of the interrelationships between teacher–student dynamics, emotional intelligence, learning enjoyment, and burnout among both undergraduate and graduate students. By employing SEM, this research transcends traditional linear analysis, offering a sophisticated framework to decipher the complex interactions and pathways among these variables. Furthermore, the selection of undergraduate and graduate students stems from the recognition of their distinct educational experiences. Undergraduates often grapple with the transitional phase into higher education, facing initial academic pressures, while graduate students encounter more specialized academic demands and professional expectations. Investigating these cohorts will enable a holistic view of how these variables operate at different educational stages, facilitating tailored interventions to support students’ well-being across various academic trajectories.

Comprehending the intricate interplay between teacher–student dynamics, emotional intelligence, learning enjoyment, and student burnout carries profound implications for educational practices and student well-being. By uncovering these relationships and mechanisms, educators and policymakers can craft targeted interventions to diminish the risk of student burnout and foster enriching educational experiences. This research endeavors to expand the existing body of knowledge on student well-being, guiding future efforts to enhance the educational journey for students across diverse educational settings.

## Literature review

### Student burnout

The term “burnout,” once confined to the realm of business, has permeated various fields, including academic settings (Li et al., 2021; Madigan and Curran, 2021). In its general sense, burnout is defined as an overwhelming state of exhaustion and fatigue resulting from the prolonged and intense depletion of personal resources under the pressures of life (Robins et al., 2018). Recognized as a significant mental health concern cutting across different age groups (Asikainen et al., 2020), burnout is particularly noteworthy in the context of university life, where the elevated stress levels associated with academic challenges contribute to academic burnout, hindering an individual’s educational progress (Liu et al., 2021; Liu, 2023).

From the existing literature, it is evident that burnout encompasses three distinct dimensions: exhaustion, cynicism, and feelings of inadequacy in the academic context (Bask and Salmela-Aro, 2013). Without appropriate measures and support initiatives, burnout can lead to a spectrum of problems, from academic underachievement to the risk of dropping out (Lackritz, 2004; Kim et al., 2018). Therefore, understanding the constituents of burnout and the contributing factors is of paramount significance, especially concerning academic development and student well-being (Madigan and Curran, 2021).

Initially, burnout was defined as a form of psychological distress primarily experienced by professionals in human services roles (Maslach, 1982). However, this definition has evolved to encompass individuals facing work-related stress in diverse professional settings (Leiter and Maslach, 2003). Furthermore, considering the demanding nature of academic pursuits, encompassing tasks such as assignments, research, lectures, and examinations, students too encounter a form of occupational stress (Maslach et al., 2001; Tang et al., 2021).

In the context of this study, we introduce the concept of “learning burnout.” This is a detrimental psychological condition stemming from cumulative or chronic stress related to learning in higher education (Loi and Pryce, 2022). It is characterized by feelings of exhaustion, cynicism, and inefficacy, significantly impacting students’ emotional, cognitive, and physical well-being during learning activities (Schaufeli et al., 2002). While burnout among teachers has received due attention due to their pivotal role in delivering quality education, limited research has focused on burnout among EFL learners (Jahedizadeh et al., 2016; Li et al., 2021; Liu et al., 2021; Wang, 2022). Notably, there has been a relative dearth of studies exploring the predictors of burnout among EFL learners.

In recent years, the necessity to investigate specific factors contributing to burnout among EFL learners has gained prominence. The EFL learning environment presents unique challenges and stressors, including language proficiency requirements, cultural adaptation, and the pressure to excel academically. These distinctive demands can heighten the risk of burnout among EFL students (Li et al., 2021; Liu et al., 2021). However, despite the evident importance of understanding burnout in this context, the literature remains somewhat limited in its exploration of the predictors of burnout among EFL learners. This knowledge gap underscores the significance of our study, which delves into the roles of teacher–student relationships, student learning enjoyment, and emotional intelligence in shaping burnout levels among Chinese EFL students. By addressing this research gap, we aim to provide valuable insights that can inform strategies and interventions to mitigate burnout and enhance the overall well-being and academic performance of EFL learners in the Chinese educational context.

### Teacher–student relationships

Teacher–student relationships constitute the bedrock of the educational experience, exerting significant influence over both academic and emotional well-being (Longobardi et al., 2016; Roorda et al., 2017). These relationships are marked by a unique dynamic, shaped by an inherent imbalance of authority and responsibility within the academic context (Frymier and Houser, 2000). Effective communication is pivotal in establishing and nurturing these connections, despite the distinctive attributes that set them apart from other relationships (Pianta et al., 2012; Taxer et al., 2019).

While the impact of Teacher–Student Relationships (TSR) on student and teacher outcomes is well-documented, a smaller body of literature has delved into its effects on learners, underscoring the need for further investigation in this field (Veldman et al., 2013; Longobardi et al., 2019). Studies have demonstrated a robust connection between TSR and teacher burnout, emphasizing the far-reaching implications of these relationships (Rodríguez-Mantilla and Fernández-Díaz, 2017; Zhang et al., 2023). A case study conducted by Lee and Schallert (2008) showcased the influential role of TSR on student composition improvements, highlighting the power of trust and rapport in the learning process. Additionally, Ma et al. (2018) found that positive TSR can elevate students’ English proficiency by fostering self-efficacy and promoting effective learning strategies, reinforcing the critical role of TSR in shaping both student and teacher experiences. Understanding the intricate dynamics of TSR is essential for creating a supportive and conducive learning environment for EFL students.

Central to teacher–student relationships is the establishment of trust and mutual respect in the classroom, significantly influencing students’ learning experiences (Pianta et al., 2012; Shakki, 2022). When students perceive their teachers as approachable, caring, and genuinely interested in their success, it cultivates a sense of trust that positively impacts their learning process (Roorda et al., 2011; Lei et al., 2016; Derakhshan et al., 2022). These positive relationships contribute to heightened learning enjoyment, as students feel more at ease expressing themselves and taking risks in their educational pursuits (Taxer et al., 2019).

Furthermore, these relationships are intertwined with students’ academic self-efficacy (Zhou et al., 2020). The belief that teachers are invested in their learning and genuinely care about their progress bolsters students’ confidence in their academic capabilities (Chen et al., 2021; Derakhshan, 2022). Emotional support from teachers, including encouragement and understanding, profoundly influences students’ emotional well-being and their level of enjoyment in the learning process (Pianta and Stuhlman, 2004). Students who perceive their teachers as supportive are more likely to experience a sense of belonging, ultimately enhancing their enjoyment in the learning process (Frenzel et al., 2009).

Conversely, negative teacher–student relationships marked by conflict, lack of support, or a sense of injustice can contribute to student burnout (Contreras et al., 2022). Burnout, characterized by prolonged emotional, mental, and physical exhaustion due to stress, is a significant concern among EFL students, particularly in the face of academic pressures and societal expectations (Li et al., 2021). In the context of EFL education in China, where students grapple with unique challenges related to language acquisition and cultural adaptation, the quality of teacher–student relationships becomes especially critical (Fu, 2023). A supportive and nurturing relationship with the teacher can help alleviate some of these challenges, contributing to a positive learning environment (Wu et al., 2023).

Several studies have delved into the impact of teacher–student relationships on burnout and well-being among students. Teuber et al. (2021) conducted a study focused on Chinese adolescents, highlighting the significance of the teacher–student dynamic and personal resources in understanding educational burnout. Additionally, Gastaldi et al. (2014) examined the impact of stress and burnout on the relationships between educators and pupils, emphasizing the reciprocal nature of these associations. Kim et al. (2018) employed a meta-analytical approach to investigate the function of social support in alleviating student burnout, providing valuable insights into the relationship between social support and student exhaustion.

Moreover, Romano et al. (2021) conducted longitudinal research to understand the repercussions of students’ perceptions of educators’ emotional support on various dimensions of academic burnout. Their work provided invaluable longitudinal data for evaluating how shifts in teacher support may influence student exhaustion over time. Together, these studies offer a comprehensive perspective on the intricate interplay between teacher–student relationships, social support, and individual resources in the context of school burnout among students. These findings collectively contribute to our understanding of the multifaceted factors that influence students’ well-being and their experiences in educational settings.

In essence, teacher–student relationships wield profound influence over students’ learning enjoyment and burnout levels within the realm of EFL education. Trust, support, and mutual respect in these relationships not only enhance emotional intelligence but also foster learning enjoyment while mitigating burnout. A nuanced understanding of these interconnected variables is imperative for enhancing the overall educational experience of Chinese EFL students.

### Learning enjoyment

The emergence of the “emotional turn” in the field of EFL research, coupled with the increasing influence of positive psychology, has brought emotions, particularly positive ones, to the forefront in the language learning process (Dewaele et al., 2018; Botes et al., 2022). Emotions, these affective experiences intricately woven into language learning activities and outcomes, are now recognized as a pivotal factor in education. The teaching profession is increasingly acknowledged as emotionally demanding (Hargreaves, 2001), with the classroom viewed as a charged environment (Pekrun and Linnenbrink-Garcia, 2014). While prior studies predominantly explored the impact of negative emotions on second language (L2) learning (Dewaele et al., 2023), recent inquiries, guided by positive psychology principles, have shed light on the transformative potential of positive emotions in this context (Dewaele et al., 2018).

Drawing upon the broaden-and-build theory, Foreign Language Enjoyment (FLE) and other positive emotions possess the inherent capacity to alleviate the detrimental effects of negative emotions and anxiety by expanding students’ momentary cognitive repertoires, enhancing essential cognitive resources, and bolstering their resilience (Fredrickson, 2001). FLE, acknowledged as a prominent positive sentiment in L2 education (Botes et al., 2022; Fathi and Hejazi, 2023), is characterized as an activating and task-focused emotion (Pekrun, 2006). Numerous studies underscore the central role of FLE in yielding highly favorable educational outcomes, including heightened engagement, increased willingness to communicate (WTC), enhanced L2 proficiency, and reduced L2 anxiety (e.g., Shen, 2021; Botes et al., 2022; Fathi et al., 2023). Additionally, FLE is intimately linked to learners’ creativity, self-efficacy, and self-assessed competence (Dewaele and Dewaele, 2020; Derakhshan and Fathi, 2023). In this context, FLE plays a pivotal role in enabling learners to immerse themselves more deeply in language input by reducing stress levels and fostering a willingness to take risks in the L2 acquisition process (White, 2018; Mohammad Hosseini et al., 2022).

A thorough examination of the academic literature reveals a body of research that investigates the interplay between the enjoyment of learning and its repercussions on student burnout within various educational settings. For instance, Li (2022) delved into the concept of classroom enjoyment and its correlation with disengagement and burnout among students studying EFL. The research underscores the pivotal role played by classroom enjoyment in alleviating disengagement and burnout. Similarly, Li et al. (2021) made a valuable contribution by conceiving and measuring burnout specifically in the context of foreign language learning among Chinese EFL students. Their work laid the foundation for understanding the unique manifestations of burnout in this specific context.

Ljubin-Golub et al. (2020) directed their attention to the experience of “student flow” and its association with burnout, examining how teacher autonomy support and student autonomous motivation influence these dynamics. Their study brought to light the significance of teacher support and student motivation in the context of burnout and the flow state. Jahedizadeh et al. (2016) delved into the factors of demotivation, perceptions of classroom activities, and mastery goals among EFL learners, seeking to predict language achievement and burnout. This research provided valuable insights into the intricate relationships between learner motivation, classroom experiences, and burnout. Furthermore, Karimi and Fallah (2021) explored academic burnout, feelings of shame, intrinsic motivation, and teacher support among EFL learners. They employed a structural equation modeling approach, which illuminated the complex connections among these variables and their impact on student burnout.

In combination, these studies offer a holistic understanding of how the enjoyment of learning and its associated factors influence student burnout within the sphere of foreign language education. They underscore the significance of classroom experiences, motivation, teacher support, and psychological factors in mitigating or exacerbating burnout in the context of EFL and language learning.

### Emotional intelligence

Emotional Intelligence (EI), encompassing the ability to perceive, understand, manage, express, and employ emotions, holds substantial relevance in personal and educational contexts (Parker et al., 2011; Llamas-Díaz et al., 2022). This multifaceted construct profoundly influences diverse aspects of an individual’s life. In educational contexts, EI has garnered attention for its positive impact on academic performance and overall well-being (Petrides et al., 2004; Allen et al., 2014; Derakhshan and Zare, 2023). There are two widely recognized forms of EI: Trait Emotional Intelligence (TEI) and Ability EI. TEI involves self-perceptions and tendencies regarding emotions, typically assessed through self-report measures (Petrides et al., 2004). Conversely, Ability EI pertains to actual emotional skills and is assessed through performance tasks resembling cognitive ability tests (Petrides and Furnham, 2001). Both facets contribute to an individual’s emotional aptitude and competence. TEI provides insights into how individuals perceive and manage their emotions, while Ability EI pertains to their practical skills in navigating emotional challenges.

In the field of L2 research, scholars have explored the implications of EI for language learning outcomes and the emotional experiences of learners, reflecting what has been termed the “emotional turn” in the field (Dewaele et al., 2018; Greenier et al., 2021). Dewaele et al. (2008) found an inverse relationship between TEI and language anxiety, indicating that higher TEI was associated with lower levels of language anxiety. Similarly, Shao et al. (2013) reported that higher TEI levels were linked to reduced Foreign Language Anxiety (FLA) and greater EFL achievement among Chinese undergraduate students. These findings emphasize the profound influence of emotional intelligence on language learning experiences. Li’s (2020) research with Chinese senior high school students further revealed a positive connection between TEI and L2 achievement and enjoyment. Higher levels of TEI were associated not only with improved academic performance but also with enhanced enjoyment and satisfaction in the language learning process. Additionally, an intervention study by Li and Xu (2019) targeting TEI among Chinese secondary students highlighted the significant impact of TEI on L2 emotions. It not only enhanced TEI but also elevated Foreign Language Enjoyment (FLE) while diminishing Foreign Language Anxiety (FLA). This intervention demonstrates the malleability of TEI and its potential to positively influence the emotional experiences of language learners.

TEI, encompassing 15 components clustered into four categories (self-control, well-being, sociability, and emotionality), offers valuable insights into human dispositions and self-perceptions regarding emotions (Petrides et al., 2007). Well-being reflects an individual’s overall contentment and life satisfaction, while self-control involves the ability to regulate impulses and external stressors. Emotionality pertains to emotional awareness, sensitivity, and the capacity to form interpersonal relationships (Petrides and Mavroveli, 2018). In the context of foreign language learning, TEI assumes a pivotal role in helping learners manage stress and anxiety, facilitate effective communication, and cultivate positive attitudes toward the target language and culture (Dewaele et al., 2008). The interplay between TEI and various facets of L2 performance has been substantiated by research, encompassing achievements in writing and oral fluency (Shao et al., 2013). Chen and Zhang (2022) further revealed TEI as a significant predictor of speaking and listening performance, though not of reading and writing. Additional investigations have explored the associations between TEI and emotional variables such as FLA and FLE. Dewaele et al. (2008) identified a negative correlation between TEI and FLA, suggesting that individuals with higher TEI experience lower levels of language anxiety. This research underscores the mediating role of emotional intelligence in shaping language learning experiences.

Li (2020) contributed to this discourse by establishing a modest to moderate relationship between TEI, FLE, self-rated English proficiency, and actual English performance among Chinese high school students. Moreover, FLE was identified as a mediator in the relationship between TEI and language performance and proficiency. These findings suggest that emotional intelligence, particularly in the form of TEI, influences learners’ emotional experiences during FL learning, impacting their language proficiency and satisfaction.

A wealth of research has explored the connection between emotional intelligence and student burnout across diverse educational environments and student cohorts. Loi and Pryce (2022) conducted a study focusing on university students, probing into the function of mindful self-care as a mediator in the relationship between emotional intelligence and burnout. Their research underscores the pivotal role of self-care practices in mitigating the impact of emotional intelligence on student burnout. Similarly, Molero Jurado et al. (2021) delved into the mediating function of emotional intelligence in the association between academic achievement and burnout among high school students. Their findings shed light on the potential of emotional intelligence to serve as an intermediary in understanding burnout within the high school milieu. Shariatpanahi et al. (2022) embarked on research within the realm of medical education, with a specific focus on Iranian medical students. Their study accentuated the impact of emotional intelligence on diverse facets of burnout, providing valuable insights into the distinctive challenges encountered by medical students. Cazan and Năstasă (2015) delved into emotional intelligence, life satisfaction, and burnout among university students. Their investigation contributed to a more comprehensive comprehension of the connections among these variables in the university setting. Furthermore, Blanchard et al. (2021) concentrated their efforts on clinical year medical students and explored the intricate interplay between emotional intelligence, burnout, and professional fulfillment. This study delved into the specific context of clinical training, offering invaluable insights into the relationship between emotional intelligence and burnout in the field of medical education.

These studies collectively offer a holistic perspective on the role of emotional intelligence in student burnout, considering diverse educational levels and contexts. They shed light on the mediating factors, unique challenges, and potential protective mechanisms linked to emotional intelligence in the realm of student well-being and burnout.

## The hypotheses

**H1**: teacher–student relationship is negatively related with student burnout

Positive teacher–student relationships play a fundamental role in creating a conducive learning environment. These relationships offer emotional support, foster trust, and provide a sense of belonging (Hamre and Pianta, 2001; Kim et al., 2018). Studies have consistently demonstrated that positive relationships between teachers and students lead to increased academic engagement, reduced behavior problems, and a more positive overall learning experience (Gastaldi et al., 2014; Romano et al., 2021; Teuber et al., 2021). Additionally, research has shown that students in such supportive environments experience lower levels of stress, which is a significant contributor to burnout (Pianta, 1999).

**H2**: Emotional intelligence is negatively associated with student burnout

Emotional intelligence, defined by Salovey and Mayer (1990), encompasses a set of abilities to understand and manage emotions effectively. Individuals with high emotional intelligence are equipped to cope with stressors and challenges, which are key components of emotional exhaustion and burnout (Maslach et al., 2001). Empirical evidence consistently supports the negative association between emotional intelligence and burnout (Cazan and Năstasă, 2015; Blanchard et al., 2021; Molero Jurado et al., 2021; Loi and Pryce, 2022; Shariatpanahi et al., 2022).

**H3**: Learning enjoyment is negatively connected with student burnout

The Self-Determination Theory (SDT) posits that when individuals find activities enjoyable and aligned with their values, they are more likely to be intrinsically motivated (Ryan and Deci, 2000; Deci and Ryan, 2008). Learning enjoyment is a key driver of this intrinsic motivation. In contrast, burnout is associated with detachment and reduced engagement, which are antithetical to the principles of intrinsic motivation (Maslach et al., 2001). Empirical research affirms this hypothesis, illustrating that students who experience higher levels of enjoyment in their learning endeavors tend to exhibit lower levels of burnout (Jahedizadeh et al., 2016; Ljubin-Golub et al., 2020; Karimi and Fallah, 2021; Li et al., 2021; Li, 2022).

**H4**: Emotional intelligence mediates the relationship between teacher–student relationship and student burnout

Positive teacher–student relationships serve as a fertile ground for emotional intelligence development in students, as they witness and learn effective emotional regulation skills through interaction and observation (Chamizo-Nieto et al., 2021). Higher emotional intelligence, in turn, empowers students with the ability to navigate their emotions effectively, potentially acting as a buffer against the development of burnout (Blanchard et al., 2021; Loi and Pryce, 2022).

**H5**: Emotional intelligence mediates the relationship between learning enjoyment and student burnout

Students with higher emotional intelligence exhibit superior emotional regulation skills, enabling them to maintain a sense of enjoyment and engagement in their learning experiences (Li, 2020). This emotional resilience, in turn, may act as a protective factor against the onset of burnout (Brackett et al., 2010; Li and Xu, 2019). Therefore, it is theoretically plausible to suggest that emotional intelligence may serve as a mediator in the relationship between learning enjoyment and student burnout.

## Method

### Participants and procedure

The data collection for this study spanned from January to April 2022. For this study, a total of 806 male and female EFL university students were selected, representing a diverse range of academic disciplines, from four universities across various regions of China. The participants exhibited a mean age of 21.15 years, with a standard deviation of 1.86 years, indicating a wide range of ages within the sample. A substantial proportion of the participants were pursuing either Master’s or undergraduate degrees, reflecting the diverse academic backgrounds within the cohort.

To ensure a comprehensive representation, participants were recruited from a mix of urban and suburban campuses, contributing to a rich demographic cross-section. Among the participants, 62.13% were pursuing undergraduate studies, while the remaining 37.41% were enrolled in master’s programs. The sample included 487 female participants (60.42%) and 319 male participants (39.57%). The cohort encompassed students majoring in diverse fields, with approximately 28.5% pursuing degrees in Engineering and Technology, 21% in Humanities and Social Sciences, 15% in Natural Sciences, 12% in Business and Economics, 9% in Fine Arts, and the remaining 14.5% distributed among various other academic domains such as Health Sciences, Education, and Agriculture. Table 1 provides the summary of the demographic information of the participants.

**Table 1:** Demographic information of participants.

Demographic information	Participants
Gender	
Female	487 (60.42%)
Male	319 (39.57%)
Academic Degree	
Undergraduate	501 (62.13%)
Master’s	305 (37.87%)
Academic Fields	
Engineering and Technology	230 (28.5%)
Humanities and Social Sciences	169 (21%)
Natural Sciences	121 (15%)
Business and Economics	97 (12%)
Fine Arts	72 (9%)
Other Fields	117 (14.5%)

The process of data collection involved reaching out to potential participants through official university channels, such as email notifications and online student portals. Invitations were extended to engage in the study by completing a series of structured online questionnaires. It was of utmost importance to emphasize that participation was entirely voluntary, and each participant was required to provide their informed consent by digitally signing a consent form prior to engaging in the study. Participants were allotted approximately 20 min to thoughtfully respond to the questionnaires, contributing their valuable insights to the research endeavor. This comprehensive approach to data collection aimed to ensure the robustness and representativeness of the findings.

## Instruments

### The emotional intelligence scale

The assessment of emotional intelligence utilized the Wong and Law Emotional Intelligence Scale, a well-validated instrument in the field (Wong and Law, 2002). This scale comprises four dimensions: self-emotion appraisal, others’ emotional appraisal, use of emotion, and regulation of emotion. Survey questions derived from this scale consisted of four carefully selected items for each dimension, totaling 16 questions. It primarily focuses on Trait EI, measuring typical patterns of emotional functioning. Cronbach’s alpha coefficients for each dimension were as follows: Self-emotion appraisal: *α* = 0.87, Others’ emotional appraisal: *α* = 0.81, Use of emotion: *α* = 0.91, Regulation of emotion: *α* = 0.83. The participants responded using a 5-point Likert scale ranging from “Strongly Disagree” (1) to “Strongly Agree” (5).

### The Maslach’s burnout inventory-student survey

The MBI-SS, a modified version of Maslach’s Burnout Inventory by Schaufeli et al. (2002), includes 15 items distributed across three subscales: “exhaustion,” “cynicism,” and “academic efficacy.” Cronbach’s alpha coefficients for each subscale were: Exhaustion: *α* = 0.93, Cynicism: *α* = 0.88, Academic efficacy: *α* = 0.91. Respondents rated each item on a 7-point Likert scale from “Never” (1) to “Always” (7).

### The learning enjoyment scale

Learning enjoyment was evaluated using four adapted items from Bieleke et al. (2021) Achievement Emotions Questionnaire, specifically selected to measure enjoyment in the context of EFL learning. These items were revised to align with the nuances of EFL education. Cronbach’s alpha for this scale was *α* = 0.88, reflecting high reliability. Participants rated items using a 7-point Likert scale from “Strongly Disagree” (1) to “Strongly Agree” (7).

### The teacher–student relationship scale

To measure the teacher–student relationship, the Chinese version of the Teacher–Student Relationship Scale (TSRS-C; Chu, 2006) with 18 items and three subscales – Situation, Intimacy, and Equality – was employed. Cronbach’s alpha for each subscale was: Situation: *α* = 0.80, Intimacy: *α* = 0.81, Equality: *α* = 0.84. Responses ranged from 0 (Strongly Disagree) to 4 (Strongly Agree) on a 5-point scale, indicating the quality of the teacher–student relationship.

## Data analysis

The analytical process for this study encompassed a series of methodical steps aimed at investigating the relationships among variables and examining the research hypothesis. Initially, descriptive statistics and correlation analyses were conducted utilizing SPSS 28.0. This facilitated a comprehensive understanding of the characteristics and interrelationships of the variables under scrutiny.

To rigorously assess the research hypothesis, we employed Structural Equation Modeling (SEM) with the assistance of the Amos program (version 26.0). This analytical approach commenced with an in-depth evaluation of the measurement model to affirm the construct validity of the utilized scales. Subsequently, the structural model was meticulously examined to scrutinize the postulated relationships among the variables. This comprehensive analysis enabled us to evaluate both the direct and indirect effects within the theoretical framework.

A range of fit indices were deployed to gauge the overall adequacy of the hypothesized model. Attention was paid to the *χ*^2^-goodness of fit to degree of freedom (df) ratio, with a value less than 3 indicating a commendable fit. Additionally, the Goodness of Fit Index (GFI) and the Comparative Fit Index (CFI) were employed, with values equal to or surpassing 0.90 signifying a favorable fit. The Root-Mean-Square Error of Approximation (RMSEA) and the Standardized Root-Mean-Square Residual (SRMR) were also taken into consideration as additional fit indices. Typically, an RMSEA value below 0.08 and an SRMR value under 0.10 are considered indicative of a robust model fit (Hu and Bentler, 1999; Tabachnick et al., 2013).

In our concluding phase, we assessed the gender invariance of emotional intelligence’s mediating effect through multigroup analyses. Initially, we scrutinized configural invariance, metric invariance, and scalar invariance (Cheung and Rensvold, 2002). Once scalar invariance was confirmed, we proceeded with further analysis to ascertain the potential invariance of path coefficients between genders. The assessment relied on evaluating chi-square differences (Δ*χ*^2^) and differences in the Comparative Fit Index (ΔCFI) to gauge the invariance of the tested parameters. Particularly, the presence of significant Δ*χ*^2^ and a negative ΔCFI value below −0.01 signaled the absence of invariance (Cheung and Rensvold, 2002).

## Results

To ensure the validity and robustness of our measurement model, a Confirmatory Factor Analysis (CFA) was conducted using AMOS 26.0. This analysis allowed us to investigate the proposed relationships between latent constructs and their corresponding observed indicators, as well as to assess the compatibility of our model with the dataset (Tabachnick et al., 2013). Our initial measurement model incorporated indicators for each of the four latent constructs, utilizing multiple observed indicators from their respective scales. However, the analysis of this initial model revealed that the fit indices did not meet the criteria for an acceptable model fit. Specifically, the initial model yielded the following fit indices: *χ*^2^(169) = 487, CFI = 0.915, TLI = 0.901, RMSEA = 0.078 [90% CI: 0.069, 0.086], SRMR = 0.067.

To improve construct validity and overall model fit, we engaged in an iterative refinement process. By thoroughly scrutinizing modification indices and following established guidelines, we identified potential sources of model misfit. Notably, two items from the teacher–student relationship scale displayed low factor loadings, indicating that they did not adequately represent the underlying construct. Consequently, these items were removed from the model to enhance construct validity and conceptual consistency. Similarly, two items from the burnout scale exhibited weak factor loadings. To enhance the clarity and unidimensionality of their respective constructs, these items were also excluded from the model.

Following the refinement process, the revised measurement model underwent another round of CFA analysis. The results of this analysis demonstrated a significant improvement in fit indices, indicating an enhanced model fit and improved construct validity. The fit indices for the revised model were as follows: *χ*^2^(141) = 298, CFI = 0.946, TLI = 0.942, RMSEA = 0.042 (90% CI [0.037, 0.046]), SRMR = 0.039. The comparison of fit indices between the initial and revised models unequivocally demonstrates that the refined model displayed markedly superior goodness-of-fit measures. Given this notable enhancement in fit indices and the reinforced construct validity resulting from these adjustments, we established the revised model as suitable for subsequent data analyses, including SEM. This model was employed to thoroughly examine the hypothesized relationships between the constructs, contributing to the rigor and reliability of our findings.

To assess the convergent and divergent validity of the latent constructs, we computed the Average Variance Extracted (AVE) and Composite Reliability (CR) values, following the methodology outlined by Fornell and Larcker (1981). AVE quantifies the proportion of variance explained by each construct relative to measurement error, while CR measures the internal consistency reliability of the constructs. The results are summarized in Table 2.

**Table 2:** Convergent and divergent validity.

Variables	AVE	CR	1	2	3	4
1. Teacher–student relationship	0.61	0.83	0.78			
2. Learning enjoyment	0.73	0.91	0.32	0.85		
3. Emotional intelligence	0.59	0.93	0.38	0.49	0.77	
4. Burnout	0.54	0.88	0.42	0.29	0.51	0.73

AVE, average variance extracted; CR, composite reliability. Numerical values in bold font represent the square roots of the AVE, while values outside the main diagonal indicate correlation coefficients.

Table 2 provides the AVE and CR values for each latent construct, with the square root of the AVE highlighted in bold font to indicate convergent validity. Elevated AVE values indicate that a substantial portion of the construct’s variance is accounted for by its indicators, thus confirming robust convergent validity. Moreover, CR values exceeding the 0.70 threshold are considered satisfactory, indicating strong internal consistency reliability. The results underscore robust convergent validity for all latent constructs, with the square roots of their respective AVEs comfortably surpassing the recommended threshold of 0.50.

In our assessment of divergent validity, we examined the correlation coefficients among the constructs, as thoughtfully presented in the off-diagonal cells of Table 1. Discriminant validity is established when the correlation coefficients between constructs are smaller than the square root of their corresponding AVEs. As depicted in the table, the correlation coefficients consistently fall below the square roots of the AVEs. This compelling evidence substantiates the distinctiveness and discriminant validity of the constructs.

Descriptive statistics and reliability indices for the study variables are provided in Table 3. Mean scores for the variables were as follows: teacher–student relationship (M = 3.01), learning enjoyment (M = 3.42), emotional intelligence (M = 2.98), and burnout (M = 3.82). Standard deviations (SD) indicated the amount of variability around the mean for each variable, with values ranging from 0.59 to 0.76.

**Table 3:** Descriptive statistics.

Variables	Mean	SD	Skewness	Kurtosis	Cronbach’s alpha
1. Teacher–student relationship	3.01	0.59	−0.11	0.18	0.82
2. Learning enjoyment	3.42	0.76	−0.06	0.08	0.88
3. Emotional intelligence	2.98	0.69	−0.19	0.16	0.91
4. Burnout	3.82	0.61	−0.04	0.14	0.93

Skewness values, measuring the symmetry of the distribution, ranged from −0.19 to −0.04, indicating generally symmetrical distributions. Kurtosis values, indicating the degree of peakedness in the distributions, ranged from 0.08 to 0.18, signifying moderately peaked distributions. Also, the internal consistency reliability of the scales, assessed using Cronbach’s alpha, was satisfactory for all variables, with values ranging from 0.82 for teacher–student relationship to 0.93 for burnout. These results indicate good to excellent reliability of the measurement scales employed in the study.

Table 4 displays the correlations between the study variables. The results uncover several significant relationships among these constructs. Firstly, a strong teacher–student relationship was found to be positively correlated with learning enjoyment (*r* = 0.32, *p* < 0.05) and emotional intelligence (*r* = 0.38, *p* < 0.01). Learning enjoyment also exhibited a positive correlation with emotional intelligence (*r* = 0.49, *p* < 0.01). Furthermore, emotional intelligence demonstrated positive associations with both learning enjoyment (*r* = 0.49, *p* < 0.01) and teacher–student relationship (*r* = 0.38, *p* < 0.01). This suggests that students with higher levels of emotional intelligence tended to derive more enjoyment from their learning experiences and reported stronger teacher–student relationships.

**Table 4:** Correlations among the constructs.

Variables	1	2	3	4
1. Teacher–student relationship	1			
2. Learning enjoyment	0.32*	1		
3. Emotional intelligence	0.38**	0.49**	1	
4. Burnout	−0.42**	−0.29*	−0.51**	1

*p < 0.05 and **p < 0.01.

In contrast, burnout showed negative correlations with teacher–student relationship (*r* = −0.42, *p* < 0.01), learning enjoyment (*r* = −0.29, *p* < 0.05), and emotional intelligence (*r* = −0.51, *p* < 0.01). These negative associations indicate that as levels of burnout increased, students were more likely to report weaker teacher–student relationships, reduced enjoyment in their learning, and lower emotional intelligence.

**Figure 1:**
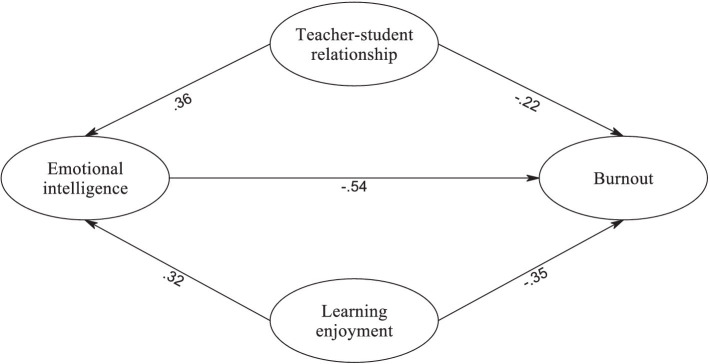
The SEM model.

Subsequently, SEM was conducted to explore the proposed relationships among the constructs. The analysis revealed that the model provided a good fit for the observed data, as evident from the following fit indices: *χ*^2^ (371) = 520.75, *p* = 0.000, CFI = 0.976, TLI = 0.972, RMSEA = 0.028, with a 95% confidence interval ranging from 0.026 to 0.032, and SRMR = 0.042. Figure 1 visually represents the path diagram depicting the expected relationships among the latent constructs. Notably, all path coefficients were determined to be statistically significant, offering empirical validation for the hypothesized associations between the variables.

Also, to assess the significance of indirect effects, bootstrapping analyses were conducted with 5,000 resamples (Hayes, 2009). The results of these bootstrapping analyses are summarized in Table 4, which provides a comprehensive overview of the direct, indirect, and total effects within the mediation analysis. Table 5 displays the outcomes of the SEM analysis, delineating the direct, indirect, and total effects among the examined variables. Firstly, the direct effect of learning enjoyment on burnout was estimated at *β* = −0.35 (95% CI [−0.42, −0.29], *p* < 0.001), signifying a significant negative relationship, indicating that higher levels of learning enjoyment were associated with lower levels of burnout. Similarly, the teacher–student relationship exhibited a direct effect on burnout with a coefficient of *β* = −0.22 (95% CI [−0.28, −0.17], *p* < 0.001), indicating a significant negative relationship. This implies that a stronger teacher–student relationship was linked to lower levels of burnout. Furthermore, emotional intelligence demonstrated a direct effect on burnout, yielding a coefficient of *β* = −0.54 (95% CI [−0.60, −0.49], *p* < 0.001), indicating a substantial negative relationship, signifying those higher levels of emotional intelligence were associated with lower levels of burnout.

**Table 5:** The results of SEM.

Path	*β*	Bootstrapped 95% CI	*p*	Effect type
Learning enjoyment → burnout	−0.35	[−0.42, −0.29]	<0.001	Direct
T–S relationship → burnout	−0.22	[−0.28, −0.17]	<0.001	Direct
Emotional intelligence → burnout	−0.54	[−0.60, −0.49]	<0.001	Direct
T–S relationship → EI → burnout	−0.19	[−0.25, −0.14]	<0.001	Indirect
Learning enjoyment → EI → burnout	−0.17	[−0.23, −0.12]	<0.001	Indirect
Learning enjoyment → burnout	−0.52	[−0.58, −0.46]	<0.001	Total
T–S relationship → burnout	−0.41	[−0.47, −0.36]	<0.001	Total

T–S relationship, teacher–student relationship; EI, emotional intelligence.

The mediation pathway from the teacher–student relationship to burnout through emotional intelligence revealed an indirect effect with a coefficient of *β* = −0.19 (95% CI [−0.25, −0.14], *p* < 0.001), indicating a significant negative relationship. This suggests that a stronger teacher–student relationship was associated with lower levels of burnout through increased emotional intelligence. Similarly, the mediation pathway from learning enjoyment to burnout through emotional intelligence exhibited an indirect effect with a coefficient of *β* = −0.17 (95% CI [−0.23, −0.12], *p* < 0.001), indicating a significant negative relationship. This implies that higher levels of learning enjoyment were linked to lower levels of burnout through increased emotional intelligence.

Overall, the total effect of learning enjoyment on burnout, considering both direct and indirect pathways, was estimated at *β* = −0.52 (95% CI [−0.58, −0.46], *p* < 0.001). Similarly, the total effect of teacher–student relationship on burnout was estimated at *β* = −0.41 (95% CI [−0.47, −0.36], *p* < 0.001).

Finally, to examine the potential gender differences in the mediating impact of teacher–student relationship, learning enjoyment, emotional intelligence, and burnout, a series of multigroup analyses were conducted. Initially, separate baseline models were assessed for each gender to ascertain the initial fit to the data. The results revealed that both the model for female participants (Ma) and male participants (Mb) exhibited a favorable fit to the data, indicating a satisfactory starting point for further gender invariance testing (see Table 6). Following this, a test for configural invariance (Mc) was conducted to verify whether the patterns of factor loadings across latent constructs were similar for both genders. Results indicated a good fit for the configural invariance model, affirming that the same latent constructs were measured across genders, laying the groundwork for subsequent analyses without substantial alterations in factor structure.

**Table 6:** Gender invariance analysis.

Model	*χ*^2^(df)	CFI	TLI	RMSEA	SRMR	Δ*χ*^2^	*p*	ΔCFI
Ma Baseline – Female	189.548 (110)	0.945	0.931	0.051	0.053	–	–	–
Mb: Baseline – Male	204.756 (110)	0.930	0.922	0.055	0.048	–	–	–
Mc: Configural Invariance	317.321 (185)	0.939	0.926	0.049	0.045	–	–	–
Md: Metric Invariance	333.669 (186)	0.940	0.928	0.048	0.052	16.348	0.45	0.001
Me: Scalar Invariance	351.209 (199)	0.939	0.929	0.047	0.050	17.540	0.38	−0.001
Mf: Constrained Model	362.435 (201)	0.938	0.927	0.047	0.053	11.226	0.56	−0.001

The subsequent phase entailed examining metric invariance (Md) by aligning factor loadings across genders. The assessment indicated a well-fitted model, and the comparison between the metric invariance model (Md) and the configurational invariance model (Mc) displayed no noteworthy differences in fit indices, supporting the concept of metric invariance. This outcome underscored the similarity in relationships between latent constructs across genders.

Further, scalar invariance (Me) was explored, equating item intercepts across genders. The model exhibited a satisfactory fit, and comparison with the metric invariance model (Md) revealed no substantial discrepancies in fit indices, confirming scalar invariance. This step involved assessing whether observed variables’ means were comparable for male and female participants. Results highlighted a fitting model, and the non-significant change in chi-square (Δ*χ*^2^ = 17.540, *p* = 0.38, ΔCFI = −0.001) confirmed the establishment of scalar invariance. It suggested that not only were the relationships consistent across genders but also the means of observed variables were alike.

Lastly, a constrained model (Mf) was employed to scrutinize equality constraints on path coefficients among teacher–student relationship, learning enjoyment, emotional intelligence, and burnout across genders. The analysis unveiled a well-fitted model, and comparison with the scalar invariance model (Me) showed no substantial differences in fit indices. The insignificant change in chi-square (Δ*χ*^2^ = 11.226, *p* = 0.56, ΔCFI = −0.001) indicated invariant associations among these constructs across male and female EFL students (see Table 6).

## Discussion

The primary aim of this study was to investigate the interplay between teacher–student relationships, learning enjoyment, emotional intelligence, and student burnout among Chinese EFL students. Specifically, we sought to explore the relationships between these variables, their implications for student well-being, and the mediating role of emotional intelligence in these associations.

Consistent with prior research (Gastaldi et al., 2014; Kim et al., 2018; Romano et al., 2021; Teuber et al., 2021), our study revealed a negative association between teacher–student relationships and student burnout. This finding aligns harmoniously with attachment theory’s premises (Bowlby, 1980). Attachment theory posits that emotional bonds with significant individuals influence emotional regulation and overall well-being (Bowlby, 1980). In the educational context, teachers often serve as crucial attachment figures, offering emotional support and a sense of security (Pianta, 1999). Positive teacher–student relationships act as sources of emotional security, effectively reducing stress and emotional exhaustion – two critical components of burnout (Maslach et al., 2001; Teuber et al., 2021). In contrast to previous studies, our research delves into the distinctive cultural landscape inherent in Chinese EFL education (Shang and Xie, 2020). Within the tapestry of Confucian-influenced societies like China, the teacher–student relationship is deeply steeped in cultural intricacies, emphasizing values of respect, hierarchy, and harmony (Tan and Chee, 2005). This cultural backdrop not only offers emotional support but profoundly influences students’ emotional regulation in alignment with collectivist ideals and filial piety (Chao, 1994; Ho, 1994). These cultural underpinnings significantly magnify the role of teacher–student relationships in providing emotional security and shed light on how cultural values shape emotional dynamics within educational settings, impacting susceptibility to burnout.

Furthermore, our findings resonate with previous research demonstrating that positive teacher–student relationships correlate with heightened student engagement (Hamre and Pianta, 2001; Li, 2018). Engaged students tend to exhibit intrinsic motivation and a strong sense of purpose, serving as protective factors against burnout (Pianta et al., 2012). When students feel emotionally connected to their teachers, they actively participate in the learning process, thereby diminishing the feelings of detachment often associated with burnout (Kim et al., 2018). Additionally, these positive relationships are linked to greater student autonomy and self-determination (Lei et al., 2016), qualities crucial in reducing the risk of burnout, as students who feel in control of their learning experiences are less prone to emotional exhaustion (Romano et al., 2021). In China, where societal emphasis on academic success is conspicuous (Cao and Liu, 2023), positive teacher–student relationships may serve as catalysts for academic motivation, resonating with the cultural emphasis on diligence and educational attainment (Chen et al., 2005; Chiu and Chow, 2011). This interweaving of emotional and academic aspects within teacher–student dynamics could potentially influence burnout through culturally induced pressures for achievement and success.

Diverse cultural and disciplinary considerations within EFL education warrant attention. In this context, language acquisition intertwines with cultural understanding and identity formation (Norton and De Costa, 2018). Thus, teacher–student relationships not only aid language learning but also shape students’ emotional connection to language acquisition and cultural integration. Consequently, their impact on burnout might encompass multiple dimensions involving emotions and identity, intricately tied to language learning and cultural adaptation.

Also, our study corroborated existing research (Jahedizadeh et al., 2016; Ljubin-Golub et al., 2020; Karimi and Fallah, 2021; Li et al., 2021; Li, 2022) by revealing a negative association between learning enjoyment and student burnout. This association aligns with SDT, which posits that intrinsic motivation and engagement thrive when individuals perceive activities as enjoyable, interesting, and congruent with their values (Deci and Ryan, 2008). In stark contrast, burnout is typified by detachment, emotional exhaustion, and reduced personal accomplishment (Maslach et al., 2001). When students find their learning experiences enjoyable, they are more likely to be intrinsically motivated, which, in turn, lowers the risk of burnout (Li et al., 2021).

This finding also resonates with existing research on student engagement, where enjoyable and relevant learning experiences are linked to reduced burnout risk (Fredricks et al., 2004). Enjoyable learning experiences kindle heightened motivation and imbue a stronger sense of purpose, acting as shields against the emotional and physical exhaustion associated with burnout (Jahedizadeh et al., 2016; Ljubin-Golub et al., 2020).

Learning enjoyment and its association with burnout might also be swayed by cultural expectations regarding academic performance. Pleasurable learning experiences might function not just as intrinsic motivators but also as stress alleviators amid academic pressures, revealing how cultural values influence the interplay between learning enjoyment and burnout. Moreover, in the discipline of language learning, where cultural assimilation is crucial, the pleasure derived from learning English could extend beyond academic engagement (Yu et al., 2019). Mastery of English for Chinese EFL students involves not only linguistic proficiency but also assimilation into Western cultural norms and values (Huang et al., 2015). Hence, the relationship between learning enjoyment and reduced burnout might encompass linguistic and socio-cultural dimensions, underscoring the significance of identity formation and acculturation in EFL learning.

In addition, our study unveiled that emotional intelligence plays a mediating role in the relationship between teacher–student relationships and student burnout. Positive teacher–student relationships often function as sources of emotional support and nurturing within the educational realm (Pianta, 1999), fostering students’ emotional well-being (Gastaldi et al., 2014). In these positive relationships, students are more likely to develop heightened emotional intelligence as teachers model and guide emotional regulation (Hamre and Pianta, 2001).

This finding aligns with attachment theory’s premise that individuals form emotional bonds with significant others, impacting their emotional regulation (Bowlby, 1980). Positive teacher–student relationships effectively act as attachment figures, reducing students’ stress levels and emotional exhaustion, both crucial components of burnout (Bowlby, 1980; Maslach et al., 2001). Moreover, emotional intelligence consistently emerges as a significant factor in mitigating burnout. Individuals with higher emotional intelligence are better equipped to handle stress, manage and regulate emotions, and navigate interpersonal relationships effectively (Cazan and Năstasă, 2015; Blanchard et al., 2021; Molero Jurado et al., 2021; Loi and Pryce, 2022; Shariatpanahi et al., 2022). Emotional intelligence within the cultural framework of Chinese EFL education might be amplified by Confucian values emphasizing interpersonal harmony and emotional regulation (Chao, 1994; Ho, 1994; Cao and Liu, 2023). This intersection of cultural values with emotional intelligence implies a nuanced interplay, where emotional competencies might be deeply rooted in socio-cultural norms, affecting their efficacy in mitigating burnout among Chinese EFL students.

Similarly, emotional intelligence mediates the relationship between learning enjoyment and student burnout. When students derive enjoyment from their learning experiences, they are more likely to be intrinsically motivated and engaged in their studies (Salmela-Aro et al., 2022). Enjoyable learning experiences can bolster emotional intelligence by aiding individuals in effectively managing their emotions (Abe, 2011). As a result, students who enjoy learning tend to develop better emotional regulation skills, serving as a protective factor against burnout (Brackett et al., 2010). This finding adheres to the tenets of SDT, which emphasizes that individuals are more likely to be intrinsically motivated when they perceive activities as enjoyable and in alignment with their values (Ryan and Deci, 2000). Engaging and enjoyable learning experiences cultivate intrinsic motivation and autonomy, both crucial for mitigating the risk of burnout (Ryan and Deci, 2000). Given the well-established importance of emotional intelligence in mediating the relationship between learning enjoyment and burnout across various domains (Cazan and Năstasă, 2015; Molero Jurado et al., 2021), this finding reinforces the robustness of the theoretical framework.

Cultivating emotional intelligence through enjoyable learning experiences might echo Confucian ideals prevalent in Chinese society, emphasizing harmony, empathy, and emotional regulation (Chao, 1994; Ho, 1994). Pleasurable learning encounters within this cultural framework might not only stimulate intrinsic motivation, as suggested by SDT, but also foster emotional intelligence steeped in Confucian virtues (Tan and Chee, 2005). The interplay between learning enjoyment and emotional intelligence may be intertwined with Confucian ethical frameworks highlighting emotions’ role in fostering social harmony and interpersonal relations (Ho, 1994). Consequently, our study’s observed relationship might be influenced by cultural values, potentially indicating a unique pathway through which learning enjoyment contributes to emotional intelligence, acting as a buffer against burnout within the specific cultural milieu of Chinese EFL education.

Furthermore, the disciplinary dimension of language learning in the EFL domain adds complexity. Proficiency in English within a Chinese context not only requires linguistic skills but also entails absorption into Western cultural elements (Chen et al., 2005). This amalgamation might render the relationship between learning enjoyment, emotional intelligence, and burnout multifaceted, shaping students’ emotional competencies influenced by both linguistic assimilation and socio-cultural integration (Li et al., 2022). As students navigate the intricacies of language acquisition intertwined with cultural assimilation, enjoyable learning experiences might facilitate not only linguistic growth but also socio-cultural adaptation, thereby contributing to the development of emotional intelligence in a distinctly intertwined manner within the EFL discipline.

The implications of our study resonate profoundly within the educational sphere, presenting opportunities for educators, policymakers, and institutions to cultivate a more enriching academic environment. Our findings underscore the urgency for a transformative shift in teacher training programs. Equipping educators with the necessary tools to foster positive teacher–student relationships stands as a pivotal pathway. Prioritizing emotional support, effective communication, and guidance within this dynamic holds promise in not only enhancing student well-being, engagement, and motivation but also in fostering a more inclusive and supportive learning environment. Furthermore, our study highlights the critical need for a paradigm shift in curriculum design and educational practices. Actively cultivating learning environments that prioritize enjoyment emerges as a vital avenue. Integrating strategies that promote student engagement, autonomy, and intrinsic motivation within pedagogical approaches and curriculum design is fundamental. This entails embracing dynamic and adaptive learning methodologies, offering students opportunities for meaningful choice, and tailoring activities that resonate with their diverse interests and values. By doing so, educational institutions can create an environment that not only enhances academic performance but also nurtures holistic student development.

Moreover, our study underscores the significance of integrating components of emotional intelligence into educational frameworks. Proposing and implementing courses and programs dedicated to emotional intelligence development can empower students with indispensable emotional regulation skills. By nurturing emotional intelligence, institutions enable students to navigate the complexities of their academic journey more adeptly, preparing them to confront challenges and stressors effectively while fostering resilience and well-being. However, these implications invite further exploration in future research endeavors. Specifically, there is a compelling need for longitudinal studies that track the efficacy of implemented interventions stemming from our findings. Assessing the long-term impact of teacher training programs, redesigned curricula focusing on enjoyment, and emotional intelligence integration will provide invaluable insights into their sustained effects on student well-being and academic outcomes.

Additionally, investigating the differential impact of these interventions across diverse cultural and socio-economic contexts remains a pertinent avenue for future exploration. Understanding how these strategies interact with cultural nuances and socio-economic disparities can refine educational practices to cater to the diverse needs of students effectively. In conclusion, our study lays the groundwork for transformative shifts in educational practices by advocating for enriched teacher–student relationships, joyful learning environments, and the integration of emotional intelligence within curricular frameworks. Embracing these implications and addressing these research directions not only promises to elevate student well-being but also contributes significantly to the evolution of a more holistic and inclusive educational landscape.

However, it is crucial to acknowledge the limitations of this study. First and foremost, our reliance on self-report measures for data collection introduces potential response biases and subjectivity. While these measures capture participants’ perceptions, they are susceptible to individual interpretation and biases. Future research could significantly benefit from the inclusion of more objective measures, particularly concerning variables such as emotional intelligence. Incorporating multiple assessment methods, including observational or performance-based metrics, would provide a more comprehensive and nuanced understanding of emotional intelligence and other related constructs. Another limitation pertains to the demographic specificity and educational level targeted in this study. Our research was confined to a particular demographic group within a specific educational level, thereby cautioning against overgeneralization of the findings. To fortify the external validity of our conclusions, replication studies encompassing broader and more diverse samples across different educational levels, cultural backgrounds, and socio-economic statuses are warranted. Examining how these relationships manifest across diverse populations would enhance the generalizability and applicability of our findings in varied educational contexts.

Moreover, the cross-sectional design employed in this study restricts the establishment of causal relationships among variables. While our study provides valuable insights into associations between teacher–student dynamics, learning enjoyment, emotional intelligence, and burnout, it does not infer causation. To unravel the intricate causal mechanisms and capture the dynamic interplay between these variables over time, longitudinal studies are imperative. Longitudinal research designs would allow for the exploration of temporal relationships and elucidate how changes in one variable influence changes in others across different developmental stages. Lastly, this study did not delve into other potentially influential factors that could impact student well-being, such as socio-economic background or cultural differences. By not considering these influential factors, the study may have overlooked essential elements contributing to student burnout and emotional experiences. Future research endeavors should encompass a more comprehensive array of factors to yield a more holistic understanding of student well-being within various contexts. Understanding the nuanced interplay between socio-economic, cultural, and educational factors would enrich our comprehension of student burnout and emotional dynamics, paving the way for more tailored and effective interventions.

## Conclusion

This study has unraveled the intricate elements contributing to student burnout within educational domains. Our rigorous exploration has shed light on the significant roles of teacher–student relationships, emotional intelligence, and learning enjoyment in shaping students’ well-being and experiences within the academic sphere.

The discerned negative associations among teacher–student relationships, emotional intelligence, and learning enjoyment with student burnout reinforce the critical importance of these variables in mitigating burnout risk. These discoveries are in lockstep with established theoretical frameworks and empirical evidence, underscoring the intertwined nature of emotional and interpersonal dynamics in students’ educational trajectories. Moreover, identifying emotional intelligence as a mediator between teacher–student relationships and student burnout, as well as between learning enjoyment and student burnout, enriches our understanding of the fundamental mechanisms impacting student well-being. These mediations offer guiding principles for interventions focused on nurturing emotional intelligence, bolstering emotional regulation, and fostering positive teacher–student relationships – strategies poised to alleviate burnout risks and elevate the overall educational process.

This research advocates for the cultivation of emotionally supportive learning environments, strengthening teacher–student connections, and integrating emotional intelligence development into educational curricula. It advocates for a holistic approach to student well-being, recognizing the interconnectedness of emotional, interpersonal, and motivational factors in shaping the student’s educational trajectory. Through these insights, we emphasize the imperative of fostering a nurturing and supportive educational milieu that fosters not only academic growth but also the holistic well-being of students.

## Data availability statement

The raw data supporting the conclusions of this article will be made available by the authors, without undue reservation. Requests to access these datasets should be directed to YL, zrs070529@sina.com.

## Ethics statement

The studies involving humans were approved by School of Foreign Languages, Xinjiang Normal University, Urumqi 830017, China. The studies were conducted in accordance with the local legislation and institutional requirements. The participants provided their written informed consent to participate in this study.

## Author contributions

YL: Conceptualization, Data curation, Formal analysis, Funding acquisition, Investigation, Methodology, Project administration, Resources, Software, Supervision, Validation, Visualization, Writing – original draft, Writing – review & editing. LZ: Conceptualization, Formal analysis, Funding acquisition, Investigation, Methodology, Project administration, Resources, Validation, Visualization, Writing – original draft, Writing – review & editing.
